# Protection of rabbits against enteropathogenic *Escherichia coli *(EPEC) using an intimin null mutant

**DOI:** 10.1186/1746-6148-2-22

**Published:** 2006-06-23

**Authors:** Tim Stakenborg, Dominique Vandekerchove, Jonas Mariën, Hans Laevens, Hein Imberechts, Johan Peeters

**Affiliations:** 1 Department of Bacteriology & Immunology, Veterinary and Agrochemical Research Centre, Groeselenberg 99, 1180 Brussels, Belgium

## Abstract

**Background:**

Diarrhea and mortality resulting from infections with enteropathogenic *Escherichia coli *(EPEC) are of major economic importance in the rabbit meat industry. There is a growing need for an effective vaccine to cope with these problems and to reduce the use of antibiotics. EPEC are characterized by an attaching and effacing virulence mechanism. This is partly mediated by the intimate binding between an adhesin, called intimin, and a translocated receptor (Tir) of prokaryote origin. We constructed an intimin deletion mutant of the rabbit EPEC (REPEC) wild-type strain 97/241.6 (bio-/serogroup 3-/O15) and examined its protective capacity.

**Results:**

After verifying its complete loss of virulence, we used the attenuated strain in vaccination-challenge experiments in which complete protection against a homologous, but virulent, strain was observed. The attenuated strain was able to persist in the intestinal lumen, where it elicited an immune response against EPEC-related virulence proteins, as was shown using an EspB-specific ELISA. Despite the priming of an immune response and the generation of specific antibodies, the intimin mutant was not able to fully protect rabbits against challenges with REPEC strains of other bio-/serogroups.

**Conclusion:**

These data indicate that protection against REPEC infections is at least partly bio-/serogroup dependent and a multivalent vaccine may be needed for protection against the full range of REPEC types. Such a combination vaccine may be developed using intimin null mutants, as the latter were clearly shown to be safe and effective against homologous infections.

## Background

Enteropathogenic *Escherichia coli *(EPEC) strains are an important cause of human and animal diarrhea. In humans, EPEC are a substantial cause of infant mortality in developing countries. In rabbit production, especially newly weaned animals are highly vulnerable to EPEC. The attaching and effacing virulence mechanism of EPEC is encoded by the chromosomal locus of enterocyte effacement (LEE) [1,2]. After initial adherence, EPEC translocate their intimin receptor (Tir) and other proteins to the eukaryotic host cell by means of an LEE encoded type III secretion apparatus. Positioning of Tir in the host cell membrane makes the interaction possible with intimin, the outer membrane adhesin encoded by *eae *[3,4]. This adhesin-receptor binding leads to an intimate contact between the bacterium and the host cell, resulting in the effacement of the microvilli and the formation of actin-rich pedestals beneath the adherent EPEC [5,6].

Compared to EPEC in humans [7], only a limited number of bio-/serogroups have been described for rabbit EPEC (REPEC). While one bio-/serogroup (1+/O109) is mainly pathogenic to suckling rabbits, other REPEC are more (3-/O15, 4+/O26, 8+O103) or less (2+/O128, 2+/O132) pathogenic to weaned rabbits [8]. The economic losses in rabbit production are hard to control because many antimicrobial agents cannot be used in rabbits, while at the same time many EPEC strains are resistant to the drugs commonly used [7,9].

Although much work has been done on unraveling the molecular pathogenesis of EPEC, it is still not clear which antigens induce protection against an infection. Experimental treatment using formalin-killed REPEC as a vaccine against challenges with *E. coli *8+/O103 proved to be impractical due to the high vaccination dose and the repeated administrations that were needed. Apathogenic *E. coli *isolates that carried only the colonizing factor of the *E. coli *8+/O103 bio-/serogroup also turned out to be impractical for use as a REPEC vaccine [10,11]. Recently, an 8+/O103 REPECΔ(*tir*/*espB*)mutant was shown to protect rabbits against this highly virulent bio-/serogroup [12]. EPEC mutants defective in the intimin encoding *eae *gene do not induce reorganization of the cytoskeletal structure underneath the site of attachment, and therefore do not form pedestals [13,14]. Based on this knowledge, we constructed a REPEC mutant incapable of reversion to the wild-type due to a disruption of the *eae *gene. The safety and protective capacity of this mutant for weaned rabbits was examined in detail.

## Results

### Construction of mutant 97/241.6Δ*eae *(3-/O15)

**Figure 1:**
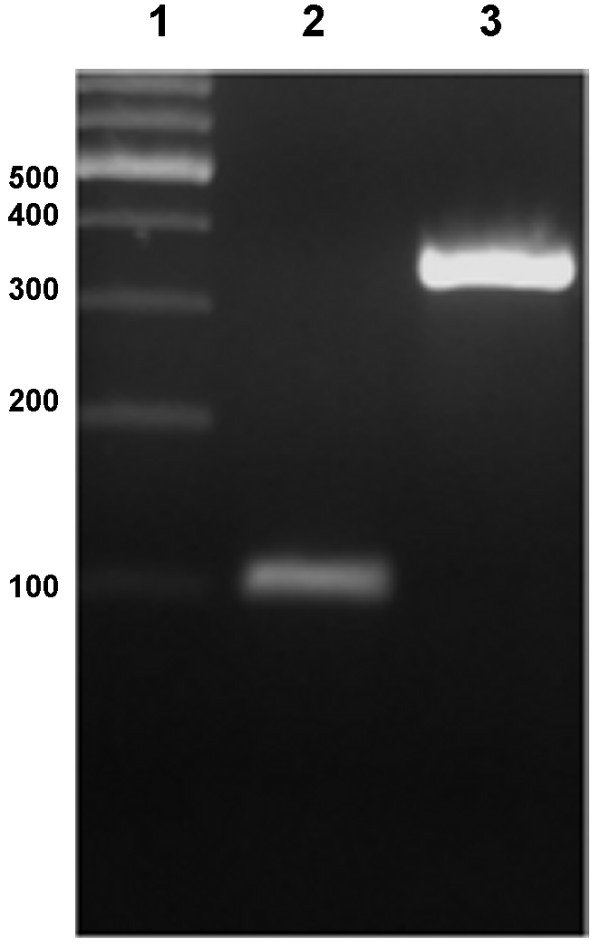
PCR amplification of part of the eae gene comprising the deleted 248 bp fragment, using primers 248 FOR and 248 REV. The amplification product of the mutant strain 97/241.6Δ*eae *is shown in lane 2, and that of the wild-type 97/241.6 strain in lane 3. A 100 bp GeneRuler (Fermentas) was used as a size-marker (lane 1).

**Figure 2:**
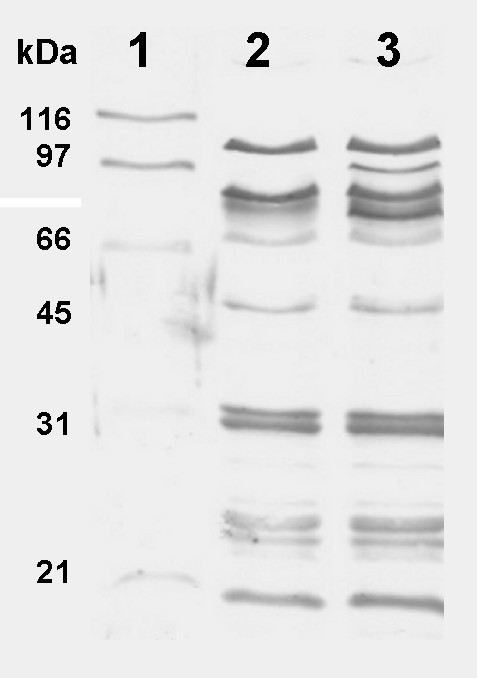
Immunoblot of the total protein fraction of strain 97/241.6Δ*eae *(lane 2) and wild-type strain 97/241.6 (lane 3). A biotinylated broad-range standard (lane 1) was used as a marker (BioRad). The intimin band (~97 kDa), marked with an arrow, is clearly present in the wild-type strain, but absent in the deletion mutant.

Only one out of 294 colonies tested had lost the 248 bp region and generated a PCR band of the expected 102 bp, compared to 350 bp for the wild-type strain (Figure 1). The deficiency in expression of intimin (~97 kDa) was verified by Western blotting using sera derived from a REPEC infected rabbit (Figure 2).

### *In vitro *brush border adhesion assay

**Figure 3:**
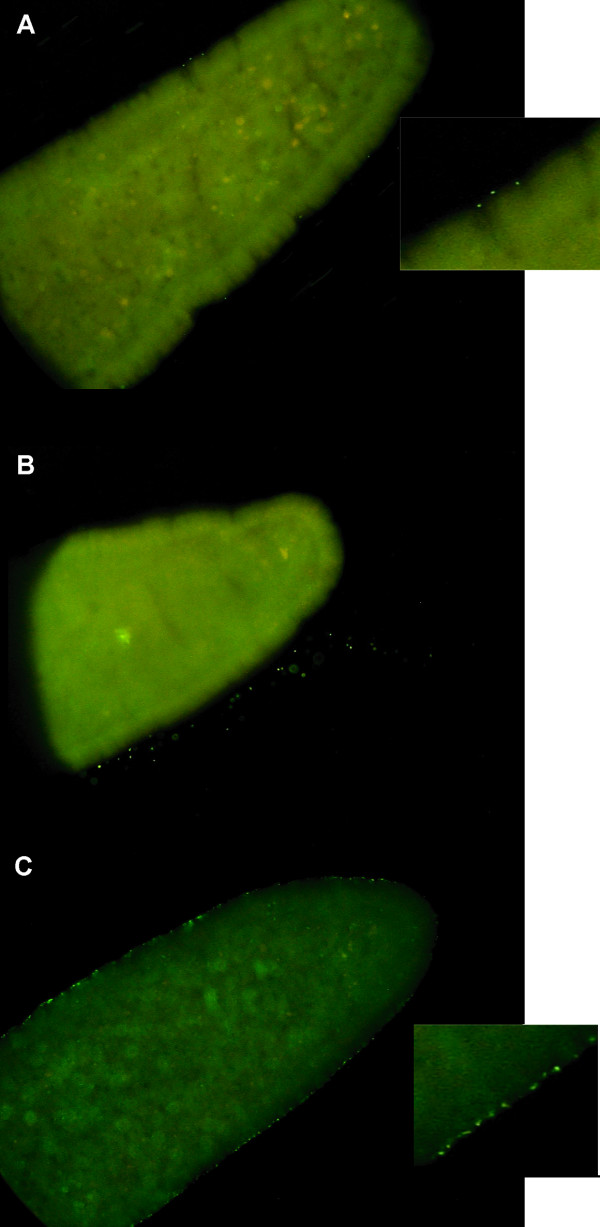
In vitro attachment of fluorescent *E. coli *strains to isolated intestinal villi. The 97/241.6Δ*eae *REPEC (A) do adhere, but only in small numbers. The insert shows three mutant bacterial cells attached to the enterocytes. DH5α strains (B) do not adhere to the intestinal villi, while B10 strains (C) are strongly adherent. The insert shows multiple *E. coli *(intense green) attached to the brush border (faint green).

The observations with the fluorescence microscope clearly demonstrated that low numbers of 97/241.6Δ*eae *bacteria were able to adhere to the brush borders of the isolated rabbit intestinal villi. Similarly low numbers of adhering bacteria were observed in the case of the wild-type 97/241.6 and 97/223.10 strains (data not shown). The DH5α strain did not adhere to the isolated villi, whereas the B10 strain adhered abundantly, as shown in Figure 3.

### Animal experiments

#### In vivo virulence of mutant 97/241.6Δ*eae*

To verify the safety of the mutant strain, one control and three groups of rabbits, which were administered different concentrations of the 97/241.6Δ*eae *strain, were examined closely over a period of four weeks.

On arrival, no rabbits were found positive for EPEC, *Clostridium spiroforme *or rotavirus. *Eimeria *spp. were detected in only small numbers in two rabbits. During the period of the study, no adverse effects due to the Baycox treatment were observed. At the end of the study, no symptoms of coccidiosis were noted at necropsy.

During the entire experiment, none of the 97/241.6Δ*eae-*inoculated rabbits showed any sign of diarrhea. Moreover, no significant differences were observed between the four groups, including the non-infected controls, for any of the parameters examined. Mutant strain 97/241.6Δ*eae *was isolated from the first day and up to three weeks after inoculation, albeit in low numbers.

#### Vaccination experiment with a homologous challenge after seven days

During an experiment lasting five weeks, the efficacy of vaccination against a homologous challenge as early as 7 days post-vaccination was examined *in vivo *using three groups of rabbits: a vaccinated-challenged group (group 1), an unvaccinated-challenged group (group 2), and a control group (group 3).

**Figure 4:**
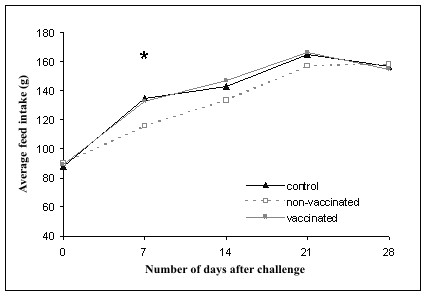
Vaccination experiment followed by challenge with a homologous strain after seven days. Average feed intake of the different groups of rabbits plotted against number of days after challenge. One group was vaccinated with strain 97/246.1Δ*eae *seven days before challenge (day -7; i.e. immediately after weaning), while both the vaccinated and the non-vaccinated group were challenged (day 0) with REPEC 97/223.10 wild-type strain. Significant differences between the non-vaccinated and the other groups are indicated with an asterisk (*:P < 0.05).

No differences were observed between the groups before challenge. After challenge, the group of non-vaccinated but challenged animals (group 2) suffered heavily from diarrhea, while no diarrhea was observed in rabbits belonging to the vaccinated group (group 1) or to the negative control group (group 3). Even though the challenge dose was low (5.4 × 10^4 ^CFU), one of the non-vaccinated animals died from colibacillosis, as was demonstrated by lesions observed at necropsy and isolation of the challenge strain from the cecal content. Non-vaccinated challenged animals systematically showed a lower weight gain compared to vaccinated and negative control animals, although the difference was not significant. The feed intake of the non-vaccinated challenged animals was significantly reduced one week after challenge compared to the negative control (*P *= 0.023) and the vaccinated rabbits (*P *= 0.043). The rabbits recovered slowly in the following weeks and the differences in feed intake disappeared. Between the vaccinated and the control group, no significant differences in feed intake were detected at any time during the experiment (Figure 4).

#### Vaccination experiment with a homologous challenge after four weeks

During an experiment that lasted eight weeks, the efficacy of vaccination against a homologous challenge after four weeks was examined *in vivo *using three groups of rabbits: a vaccinated-challenged group (group 1), an unvaccinated-challenged group (group 2), and a control group (group 3).

**Figure 5:**
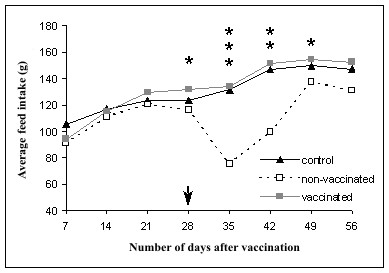
Vaccination experiment followed by challenge with a homologous strain after 28 days. Average feed intake of the different groups of rabbits plotted against day after vaccination. Vaccination with strain 97/246.1Δ*eae *was performed immediately after weaning. Both the vaccinated and non-vaccinated groups were challenged at day 28 after vaccination (arrow) with strain 97/223.10. Significant differences between the non-vaccinated and the other groups are indicated with asterisks (***: P < 0.001; **:P < 0.01; *:P < 0.05).

Before challenge, no apparent differences between the groups were observed. Even after challenge with 10^6 ^CFU of a virulent strain, no differences were observed between the vaccinated group (group1) and the negative control group (group 3). However, during the first two weeks after challenge, the animals of the non-vaccinated group (group2) showed clear signs of diarrhea and differed significantly from the vaccinated and control groups for both feed intake (*P *< 0.001, shown in Figure 5) and body weight (*P *< 0.001, results not shown).

**Figure 6:**
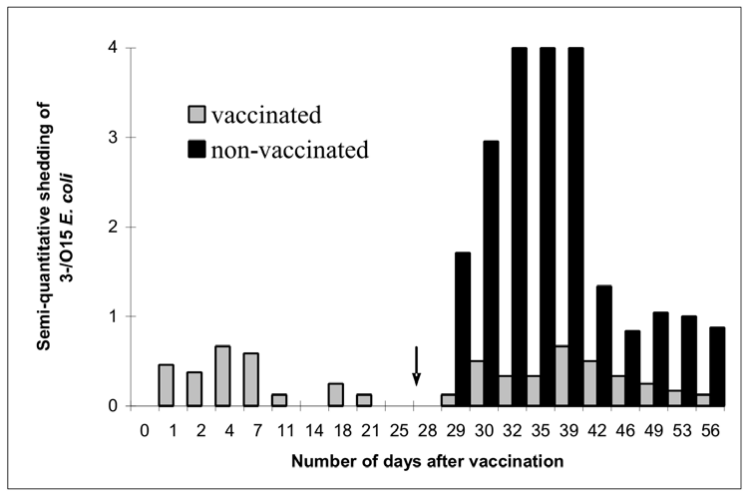
A semi-quantitative assessment of the shedding of the vaccine strain (before day 28) and wild-type strain (after day 28). The day of infection is indicated by the arrow. Grey bars represent the shedding by the vaccinated animals, black bars the shedding by the non-vaccinated rabbits (0 = no shedding, 4 = confluent colonies on agar plate).

Low numbers of the vaccine strain were shed for up to 3 weeks after vaccination with a peak at the end of the first week. After challenge, only the wild-type strain was detected, as was verified by PCR. Semi-quantitative analysis of the shedding of *E. coli *showed a clear difference between the vaccinated and the non-vaccinated groups (Figure 6). The non-vaccinated animals shed the wild-type strain in high numbers, whereas the vaccinated rabbits shed the virulent strain in only limited numbers.

#### Vaccination experiment with heterologous challenge after four weeks

**Figure 7:**
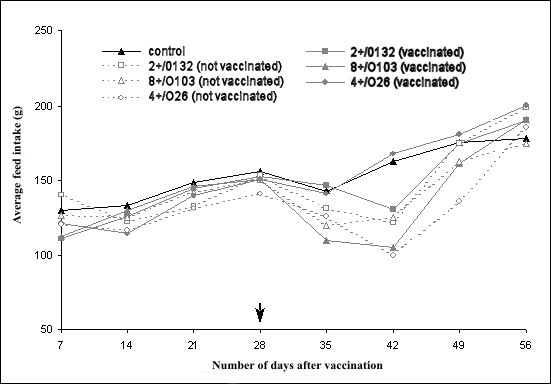
Vaccination experiment followed by challenge with heterologous strains after 28 days. Average feed intake of the different groups of rabbits plotted against day after vaccination. Vaccination with strain 97/246.1Δ*eae *(3-/O15) took place immediately after weaning. All groups, with the exception of the control group, were challenged at day 28 after vaccination (arrow) with a virulent strain of the bio-/serogroup indicated in the legend.

At the beginning of a third vaccination-challenge experiment, three groups of weaned rabbits were vaccinated with strain 97/241.6Δ*eae*. Four weeks later, these and three other groups of rabbits were challenged with heterologous virulent strains and were examined for another four weeks. A seventh group (group 1) was included as a negative control. No significant differences were detected between the groups for the observed parameters during the first four weeks. After challenge (day 28), the feed intake of the control group differed significantly from all other groups (*P *< 0.001), with the exception of group 6 (vaccinated and infected with the 4+/O26 strain) (Figure 7). The latter group also differed significantly from group 7 (non-vaccinated and infected with the 4+/O26 strain) as a function of time (*P *= 0.028). No significant differences in feed intake were observed between vaccinated and non-vaccinated groups challenged with other heterologous strains (Figure 7). Similar results were observed for weight gain (data not shown).

After challenge, diarrhea was observed in all groups except for the control group. The diarrhea scores of the two groups infected with a virulent 2+/O132 strain were alike. In both groups, two rabbits showed severe signs of diarrhea. The two groups infected with the 8+/O103 strain had similar diarrhea scores as well, with three rabbits of the non-vaccinated group (group 5) and four rabbits of the vaccinated group (group 4) showing severe diarrhea. Again, a difference was observed between the groups challenged with the 4+/O26 strain. In the non-vaccinated group (group 7), five rabbits showed severe diarrhea for more than one week. Three of these rabbits died before the end of the experiment. In the vaccinated group (group 6), only two rabbits showed mild diarrhea for only three days. In this group, none of the rabbits died.

### EspB-specific ELISA

**Figure 8:**
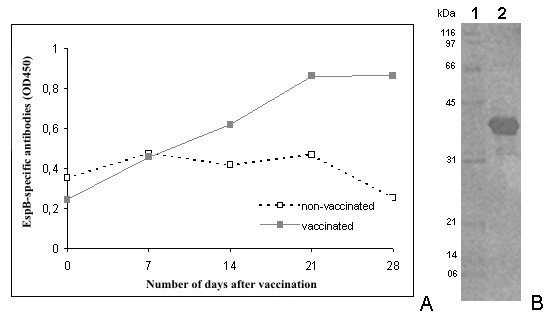
A: Detection of the EspB-specific serum antibodies of non-vaccinated and vaccinated rabbits as a function of time. Groups of 12 rabbits were used. The means were significantly different (P = 0.01) from day 14 onward. B: SDS-PAGE followed by silver-staining of a molecular weight standard (1) and purified recombinant EspB (2). The EspB band of approximately 40 kDa is clearly visible.

The concentration of the recombinant EspB solution was estimated to be around 0.7 mg purified protein per ml and was diluted to one μg per ml to coat the microtiter plates. As shown in Figure 8, the titers of EspB-specific antibodies are significantly higher from day 14 onward for the vaccinated compared to the non-vaccinated groups of the second challenge experiment (*P *= 0.01).

## Discussion

Diarrhea caused by REPEC is a major problem for rabbit production. In this study we constructed an intimin null mutant and tested its protective potential in vaccination-challenge experiments.

The safety of the live attenuated 97/241.6Δ*eae *strain was clearly demonstrated, both by the *in vivo *virulence test and by the three challenge experiments. This is in accordance with the loss of virulence for an intimin null mutant of an attaching and effacing *Citrobacter rodentium *strain [15], but in contrast to a human E2348/69Δ*eae *strain that produced signs of diarrhea in 4 out of 11 infected volunteers [13]. The latter results might be explained by the presence of additional virulence factors in human EPEC strains, such as a cytolethal distending toxin that has been described for the E2348/69 strain [16] and not for REPEC so far.

Our vaccination-challenge experiments showed that the 97/241.6Δ*eae *strain offers complete clinical protection against infection with a homologous 3-/O15 virulent REPEC strain. This was observed whether the challenge took place one or four weeks after vaccination. During both *in vivo *experiments, the rabbits of the vaccinated groups showed a significantly higher feed intake than those of the non-vaccinated groups. The lack of a significant difference between the weights of the animals during the first challenge experiment was likely due to the lower infection dose. The increase of the dose in the second challenge experiment resulted in more severe clinical signs and a significant difference in weight between the vaccinated and the non-vaccinated group. Similarly, a 4+/O26 C230Δ*eae *mutant had no remaining virulence and was also able to protect rabbits against a later infection with a pathogenic C230 wild-type strain (see Additional File 1). Moreover, a recent report showed similar results for an 8+/O103 REPECΔ(*tir*/*espB*) mutant which also targets the intimin-Tir interaction [12]. These three independent results substantiate that attenuated strains with a deletion in the genes responsible for intimate binding are avirulent and can be used to protect rabbits against infection with homologous REPEC for up to at least four weeks after vaccination. Since meat rabbits are normally slaughtered at 10 or 11 weeks of age, and since they are most susceptible to colibacillosis during the first two weeks after weaning (at the age of five to six weeks), a single vaccination at weaning may suffice to protect the animals against REPEC during their entire life span. However, further studies are needed to confirm this hypothesis since a similar vaccination experiment failed to fully protect individuals against experimental infection with a virulent human EPEC strain 70 days after vaccination [17]. Together with the decline of the local immune response over time, the high infection dose used during the latter study may have contributed to the results observed.

In an earlier study it was shown that both intimin and a REPEC adherence locus (*ral*) were required for the colonization of a 3-/O15 REPEC strain [18]. These results are in agreement with our *in vitro *adhesion studies and shedding data. Apparently, the mutant strain is able to adhere and persist in the intestine for over three weeks, albeit in low numbers. This persistence of the mutant strain was sufficient to elicit a specific immune response, as was shown by an EspB specific ELISA, and to protect the animals against colonization by a homologous pathogenic strain. Conversely, only partial protection – if any – was observed when the rabbits were challenged with heterologous REPEC strains. The 3-/O15 mutant was largely protective against a challenge with a virulent 4+/O26 strain, but failed to protect rabbits against the virulent 8+/O103 and 2+/O132 strains.

It is unlikely that the lack of cross-protection between REPEC types is the result of different subtypes of LEE encoded proteins. Different subtypes of LEE-related proteins have been described for a number of hosts [19,20], but whether this is also the case for rabbits and whether these subtypes may influence protection is questionable. Also whether or not the outer membrane lipopolysaccharides elicit a protective immune response remains to be elucidated. Many cross-reactions between polysaccharides have been described [21], but to our knowledge, no data are available about cross-reactivity between the serogroups studied [22]. Experiments in chickens with fimbrial antigens and lipopolysaccharides as potential vaccine candidates showed promising results [23]. However, a subsequent study by the same research unit [24] also showed a lack of protection against a heterologous challenge.

Colonization factors, possibly related to certain serogroups, may also explain our observed results. Several fimbriae have already been determined to enhance pathogenicity [25-27], but the relation of colonization factors to bio-/serogroup needs further study. So far, the expression of AF/R2 has only been described in 8+/O103 strains [27], while a *ral *encoded adhesin has been described for a 3-/O15 strain [25].

## Conclusion

Our experiments indicate that an *eae *deletion mutant may prove to be an effective, practical and safe vaccine candidate for protection against homologous challenges, but it cannot completely protect against heterologous virulent REPEC strains. Our study may also be helpful in the process of understanding the pathogenesis of EPEC in humans. The extensive genome and protein conservation between rabbits and primates [28] and the fact that REPEC pathogenesis shows more similarities with human EPEC infections [29,30] than do the commonly used *C.rodentium *infections in mice, make the rabbit an interesting animal model. Further studies using deletion mutants targeting other antigens may help to elucidate which factors are protective. In the mean time, the use of a combination vaccine consisting of the main REPEC bio-/serogroups may prove an effective tool in the control of colibacillosis in rabbits.

## Methods

### Bacterial strains, plasmids and media

The REPEC strains 97/241.6 (3-/O15), 97/223.10 (3-/O15), 82/90 (2+/O132), 97/110.6 (8+/O103) and 00/195.1 (4+/O26) were Belgian field isolates. REPEC B10 and RDEC-1 are described elsewhere [31]. The DH5α and Top10F' cells used in this study are commercially available (Invitrogen, UK). The 97/241.6Δ*eae *strain constructed in this study was selected for tetracycline sensitivity by the method of Bochner *et al*. [32] and was deposited under No. P-18935 on April 22, 1999 in the Belgian Coordinated Collections of Microorganisms (LMG, Ghent, Belgium).

The pRSET-B expression and pCR2.1-TOPO vectors were used according to the manufacturer's instructions (Invitrogen). The pKO3 suicide plasmid is described in detail by Link *et al*. [33].

*E. coli *were grown in Luria-Bertani Broth (LB) or on LB agar (LA) and stored at -80°C in LB containing 50% glycerol. Ampicillin (50 μg/ml) was added where appropriate. SOC medium (Sigma, Mo., USA) was used to revitalize cells after transformation. For inoculation purposes, strains were grown in Penassay broth (Difco, Md., USA).

### Construction of mutant 97/241.6Δ*eae *(3-/O15)

The sequence of the *eae *gene of strain 97/241.6 was determined (Genbank accession no. AY255520). The gene was amplified using 2 U of Expand™ polymerase (Roche Diagnostics, Switzerland) and 20 pmol of both EAE RDEC-1 FOR and REV primer (Table 1), and then ligated in a pCR2.1-TOPO vector. Next, the insert was cut out using *Bam*HI and ligated in a *Bam*HI restricted pKO3 vector, resulting in pKO3::*eae*. After ligation, an internal 248 bp region of *eae *was removed by *Pst*I digestion and self-ligation. Electroporation of the pKO3::Δ*eae *vector obtained in REPEC strain 97/241.6 was performed using 0.2 cm cuvettes in a Gene Pulser apparatus (BioRad, Ca., USA) at 200Ω ; 2.3 kV; and 25 μF. The chromosomal gene replacement was performed as described by Link *et al*. [33]. Candidate mutants were selected and tested by PCR using primers flanking the deleted 248 bp (primers 248 FOR and REV in Table 1). The loss of expression of intimin was confirmed using SDS-PAGE followed by silver staining and Western blotting by a method adapted for semi-dry transfer [34]. An SDS-PAGE broad-range standard was used as a marker (BioRad).

### Expression and purification of recombinant EspB

A set of EspB primers (Table 1), which includes the hexanucleotide recognition site of *Bam*HI and *Hind*III, was used to amplify the *espB *gene of REPEC strain 97/241.6. The resulting PCR fragment was cloned in a pCR2.1-TOPO vector and subsequently excised using *Hind*III and *Bam*HI for ligation in a double restricted pRSET-B-vector. The resulting pRSET::*espB *vector was transformed in electrocompetent Top10F' cells as described above.

To express the recombinant EspB, 50 ml of LB medium containing ampicillin was inoculated with 1 ml overnight culture of Top10F'/pRSET::*espB *cells, while 50 mmol of IPTG and 5.10^10^PFU of bacteriophage M13 were added after one and three hours of incubation at 37°C, respectively. After four hours of incubation, the cells were harvested and the recombinant EspB protein, which contains a His-tag, was purified by means of Ni^2+^-affinity chromatography (Xpress purification kit, Invitrogen). The eluates were checked for purity using silver staining and Western blotting as described above. The concentration of the recombinant EspB eluates was estimated using the micro BCA protein assay (Pierce, Il., USA) (Figure 7).

### EspB-specific ELISA

Affinity-purified recombinant EspB was coated onto 96-well microtitre plates overnight at 4°C at a concentration of 1 μg per ml coating buffer (15 mM Na_2_CO_3_, 35 mM NaHCO_3_, 3 mM NaN_3_; pH 9.6). The next day, the plates were washed three times with washing buffer (150 mM NaCl, 10 mM Na_3_PO_4_, 0.05% (v/v) Tween-20; pH7.2). After washing, the plates were blocked with coating buffer containing 5% (w/v) bovine serum albumin for one hour at room temperature. Subsequently, the plates were washed as described above and rabbit serum was added at a dilution of 1:100. The rabbit sera of the vaccinated and non-vaccinated groups of the second challenge experiment were used to monitor the EspB specific antibody response. Following incubation at room temperature for two hours, the plates were washed and incubated for one hour with biotinylated donkey anti-rabbit immunoglobulins diluted 1 to 3000 (Amersham, UK). After washing, peroxidase streptavidin conjugate (Roche Diagnostics) was added for one hour at room temperature at a dilution of 1:3000. After washing the plates four times, the bound antibodies were detected using the TMB microwell peroxidase substrate (Kierkegaard & Perry Laboratories, Md., USA). After five minutes, 1 M H_3_PO_4 _was added to stop the reaction and the optical density was measured at 450 nm.

### *In vitro *brush border adhesion assay

Intestinal villi of a six-week-old New-Zealand rabbit (Versele-Laga, Belgium) were harvested using a previously described technique [35,36]. The *E. coli *strains were FITC-labeled by incubating 10^8 ^microorganisms during 30 minutes with a 0.1 M Na_2_CO_3 _solution containing 0.5 mg FITC (Sigma) per ml. After the bacteria were washed several times with PBS containing 1% mannose, they were incubated together with the villi for one hour at room temperature. After incubation, the villi were washed five times with PBS to eliminate non-adhering bacteria. The intestinal villi were subsequently spread out on a glass slide and a fluorescence microscope was used to visualize the attached *E. coli *strains. Pictures were captured using an MDS120 camera (Kodak, NY, USA). Strain B10, expressing the AF/R2 adhesin, was used as a positive control, and strain DH5α as a negative control.

### Animal experiments

For all experiments, four-week-old, newly weaned New Zealand white rabbits (Versele-Laga) were used in accordance with the Felasa guidelines [39]. On arrival (i.e. immediately after weaning), the rabbits were screened for the presence of EPEC, *Clostridium spiroforme *and *Eimeria *spp., as described by Vandekerchove *et al*. [37]. The presence of rotavirus was examined using a commercial ELISA kit (Pathasure Enteritis ELISA kit, Vetoquinol Diagnostics, France). Rabbits testing positive for *Eimeria *spp. were treated with Baycox (Bayer, Germany) according to the manufacturer's instructions. The rabbits were housed individually and received feed pellets and water ad libitum. The daily feed intake, weight gain and diarrhea was monitored at least three times a week. The diarrhea was measured semi-quantitatively (0: no diarrhea, 1: deformed feces, 2: sticky feces, 3: confluent feces, 4: liquid feces). For all experiments, the shedding of *E. coli *was semi-quantitatively monitored (0: no colonies; 4: confluent colonies) several times a week by taking rectal swabs that were plated onto Simmons citrate agar (SCS) and/or Gassner agar (G2S) [38]. Serum was collected once a week from each rabbit. All rabbits, whether found dead or euthanatized at the end of the experiment, were necropsied.

Four animal experiments were performed: a first to determine the remaining virulence of the 97/241.6Δ*eae *mutant, and three vaccination-challenge experiments to assess its immunoprotective features.

#### *In vivo *virulence of mutant 97/241.6Δ*eae*

A total of 32 rabbits were allotted by weight and litter to four homogenous groups of 8 rabbits each. Each rabbit in all the groups received one ml of Penassay broth containing 0; 5 × 10^4^; 5 × 10^6^; and 5 × 10^8 ^colony forming units (CFU) of the 97/241.6Δ*eae *strain. The feed intake, weight gain and occurrence of diarrhea were recorded for each animal on a daily basis for a period of four weeks. The groups were statistically compared using a repeated measures general linear model (SPSS Inc., Il., USA).

#### Vaccination experiment with a homologous challenge after seven days

In the first vaccination experiment, 42 rabbits were allotted by weight and litter to three homogenous groups of 14 rabbits each. The first group was inoculated per os with one ml of Penassay broth containing a dose of 2.5 × 10^5 ^CFU of mutant 97/241.6Δ*eae*. One week later both this group and the second group were infected with 5.4 × 10^4 ^CFU of wild-type REPEC 97/223.10. The third group was used as an unvaccinated, unchallenged control group. The experiment lasted for five weeks and the weight gain, feed intake and occurrence of diarrhea were observed closely. The groups were statistically compared using a mixed procedure with the observed values of the animals before infection used as a covariate (SAS Institute GmbH, Germany). The unstructured covariance model was included in the analysis to account for correlation between the repeated measures. This covariance structure was chosen by evaluating the Akaike's Information Criterion and was preferable over the autoregressive (order 1) and the compound symmetric structure. Although the autoregressive (order 1) structure was preferable when evaluating the variance-covariance structures with the Schwarz' Bayesian Criterion, it did not have any influence on the significance level of the parameters that were evaluated.

#### Vaccination experiment with a homologous challenge after four weeks

In the second vaccination-challenge experiment, 30 animals were allotted to three homogenous groups of 12, 12 and 6 animals on the basis of weight and litter. Upon arrival, the first group was inoculated per os with one ml of Penassay broth containing 10^8 ^CFU of mutant 97/241.6Δ*eae*. Four weeks later, this group and the second group were challenged with 10^6 ^CFU of wild-type REPEC 97/223.10. The third group was used as a control group. The experiments lasted for eight weeks in total and the weight gain, feed intake and occurrence of diarrhea were observed closely. The groups were statistically compared using a repeated measures general linear model (SPSS).

#### Vaccination experiment with heterologous challenge after four weeks

The third vaccination-challenge experiment was conducted using seven homogenous groups of 9 rabbits. Upon arrival, 10^8 ^CFU of 97/241.6Δ*eae *(3-/O15) was administered orally to groups 2, 4, and 6. Four weeks later, all groups except the negative control group (group 1) were challenged with a virulent strain: groups 2 and 3 with 5 × 10^7 ^CFU of strain 82/90 (2+/O132), groups 4 and 5 with 2 × 10^7 ^CFU of strain 97/110.6 (8+/O103), and groups 6 and 7 with 10^7 ^CFU of strain 00/195.1 (4+/O26). The experiment lasted for eight weeks in total and the weight gain, feed intake and occurrence of diarrhea were observed closely. The groups were compared using a mixed procedure with the observed values of the animals before infection used as covariate as described above (SAS).

## Abbreviations

CFU colony forming units

FITC Fluoresceinisothiocyanate

G2S Gassner agar

IPTG isopropyl-beta-D-thiogalactopyranoside

LA Luria-Bertani agar

LB Luria-Bertani broth

LEE locus of enterocyte effacement

REPEC rabbit enteropathogenic *Escherichia coli*

SCS Simmons citrate agar

## Authors' contributions

TS collected most of the data and was the principal writer of the manuscript. JM participated in the construction of the mutants, while HL helped in the statistical analysis of the data. HI and JP participated in the discussion of the data, participated in proofreading and management. DV conceived the study and revised the manuscript critically. All authors made contributions and read and approved the final manuscript.

## Supplementary Material

Additional file 1 Construction of a 4+/O26 intimin null mutant and its use in a vaccination-challenge experiment. Click here for file

## Figures and Tables

**Table 1:** Primers and cycle conditions used in this study

Primer	Sequence	Number of cycles (Cycle conditions)^1^
EAE FOR	5'AAAAAAGGATCCAAAAATTACGCCGGAAGAT	30 (15" 94°C; 30" 55.4°C; and 2' 68°C)
EAE REV	5'AAAAAATCTAGAAAAAGGTAAACAGGTCTC	
		
248 FOR	5'CCACTCAAAAAACTGTCTGTTG	25 (15" 94°C; 30" 58.1°C; and 1' 72°C)
248 REV	5'TCCAGTGAACTACCGTCAAAG	
		
ESPB FOR	5'GGATCCGAATACTATTGATTATACTAAT	30 (1' 94°C; 1' 52°C; and 2' 72°C)
ESPB REV	5' AAGCTTTAAACGTATCGACCATGAT	

^1 ^All PCRs, unless stated otherwise, were performed using 3U of recombinant *Taq *polymerase, 5 μl of PCR buffer including 2 mM MgCl_2_, 0.2 mM each of dNTP and 10 pmol of both forward and reverse primer.
