# Supplementary material for: Protection of rabbits against enteropathogenic Escherichia coli (EPEC) using an intimin null mutant
Source: BMC Vet Res. 2006 Jun 23;2:22. doi: 10.1186/1746-6148-2-22 (PMC1544329; doi:10.1186/1746-6148-2-22)
Supplement: Additional file 1 — Construction of a 4+/O26 intimin null mutant and its use in a vaccination-challenge experiment. [file 1746-6148-2-22-S1.doc]

**Supplemental data**

Evaluation of an intimin mutant (C230Δ*eae*) of bio/serogroup 4+/O26

# Construction of mutant C230Δ*eae* (4+/O26)

## **Methods**

A direct PCR mutagenesis was performed as originally described by Datsenko & Wanner [1]. In short, C230 cells were made electrocompetent, transformed with the pKD46 plasmid and grown on LA plates containing ampicillin. A single colony was grown in the presence of ampicillin and 0.2% L-arabinose and made electrocompetent after confirming the presence of the pKD46 plasmid by PCR. The kanamycin resistance gene (*kan*) of plasmid pKD4 was amplified using primers DM FOR and DM REV (see Table 1) and 1 μl of the PCR product was directly used to transform the electrocompetent C230(pKD46) cells. These cells were incubated overnight at 30 °C on LA plates containing kanamycin. The pCP20 plasmid was transformed in one selected colony to eliminate the kanamycin resistance gene. After growing overnight at 30°C, the cells were placed on non-selective LA plates at 43 °C. The loss of resistance genes was confirmed by means of replica plating. A pure C230Δ*eae* colony was picked up, grown in LB and stored at -80° in the presence of 50% glycerol. The sequence of the scar (i.e*.* the remaining sequence after loss of the kanamycin resistance gene) was determined.

Results

The PCR results, using the primers PET FOR and PET REV to amplify the entire *eae* gene, showed a successful deletion of the *eae* gene (Fig. 1). Sequence analysis confirmed these results and identified a remaining scar of 84 bp.

# Animal experiment

Methods

## A vaccination-challenge experiment was performed in the same way as the other animal experiments performed in the study. A total of 15 New-Zealand white rabbits were allotted to three homogenous groups of five animals based on their weight and litter. On arrival (i.e. directly after weaning), the third group was inoculated per os with 1 ml Penassay broth containing 10^9^ CFU of the mutant strain C230Δeae. Four weeks later this and the second group were challenged with 10^8^ CFU of the wild-type strain C230. The first group was used as negative control group. The experiment lasted for 8 weeks and the weight gain, feed intake and occurrence of diarrhea were observed closely. The groups were statistically compared using Proc MIXED (SAS), with the observed values of the animals before infection being used as a covariate. An autoregressive variance-covariance structure was included in the analysis to account for correlations between the repeated measures.

Results and discussion

Throughout the entire duration of the experiment, no significant differences were detected between the vaccinated group and the control group. As shown in Figure 2, the average feed intake of the non-vaccinated group was clearly lower than in the two other groups (*P* < 0.001). Similar results were observed for weight gain (*P* < 0.05; data not shown). Moreover, four out of five rabbits of the non-vaccinated group showed clear signs of diarrhea. Three of these rabbits died with lesions of colibacillosis, whereas no mortality occurred in the other two groups.

These results show that rabbits vaccinated with an intimin null mutant are fully protected against a challenge with the corresponding wild-type strain.

**References**

1. Datsenko KA, Wanner BL**: One-step inactivation of chromosomal genes i*n Escherichia co*li K-12 using PCR product**s*. Proc Natl Acad Sci US*A 2000**, 9**7:6640-6645.

**Table 1 -** Primers and cycle conditions used during this study

| Primer | Sequence^1^ | Number of cycles (Cycle conditions)^1^ |
| --- | --- | --- |
| DM EAE FOR | 5'TGAACTAAAATGTCCCCGGACGCGACTAAAAGCAACACGACCGATGACAAGGCTCTAAA**GTGTAGGCTGGAGCTGCT** | 25 (30" 94°C; 30" 53,9°C; and 2' 72°C) |
| DM EAE REV | 5'GCAATTACAATACTACCCGGTGCATTAATTGTGTATGTCACACTCTGATTATCACCAG**CATATGAATATCCTCCTTA** |  |
|  |  |  |
| PET FOR | CCCATAACATGATTACTCAT | 25 (30" 94°C; 30" 51,5°C; and 2' 72°C) |
| PET REV | CGGGATTTGAGATGTAATTA |  |

^1^ The sequence of primers DM EAE FOR-REV indicated in bold are complementary to the antibiotic resistance gene of the pKD4 plasmid, while the underlined sequences are complementary to the *eae* gene.


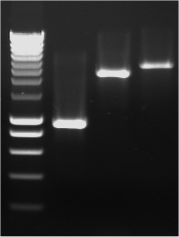


1 2 3 4

3000

2500

2000

1500

1000

800

600

400

200

**Figure 1 - Visualization of the PCR product using primers PET FOR and PET REV of the C230Δ*eae* strain (lane 2), the C230::*kan* (lane 3) and the C230 wild-type strain (lane 4). The SmartLadder (Eurogentec) was used as a size standard (lane 1).**

**Figure 2 - Average feed intake of the different groups of rabbits during the vaccination-challenge experiment with the C230Δ*eae* and the corresponding wild-type strain. Groups 2 and 3 were challenged on day 0. Only group 3 was vaccinated four weeks before challenge (day –28). Group 1 was used as a negative control.**
